# What matters in mental health care? A co-design approach to developing clinical supervision tools for practitioner competency development

**DOI:** 10.1017/gmh.2022.53

**Published:** 2022-10-21

**Authors:** Bettina Böhm, Gregory Keane, Myriam Karimet, Miguel Palma

**Affiliations:** Médecins Sans Frontières, Paris, France

**Keywords:** Clinical supervision, competency-based supervision, humanitarian settings, mental health, mhGAP, task-sharing

## Abstract

**Background:**

Specialised mental health (MH) care providers are often absent or scarcely available in low resource and humanitarian settings (LRHS), making MH training and supervision for general health care workers (using task-sharing approaches) essential to scaling up services and reducing the treatment gap for severe and common MH conditions. Yet, the diversity of settings, population types, and professional skills in crisis contexts complicate these efforts. A standardised, field tested instrument for clinical supervision would be a significant step towards attaining quality standards in MH care worldwide.

**Methods:**

A competency-based clinical supervision tool was designed by Médecins Sans Frontières (MSF) for use in LRHS. A systematic literature review informed its design and assured its focus on key clinical competencies. An initial pool of behavioural indicators was identified through a rational theoretical scale construction approach, tested through waves of simulation and reviewed by 12 MH supervisors in seven projects where MSF provides care for severe and common MH conditions.

**Results:**

Qualitative analysis yielded two sets of competency grids based on a supervisee's professional background: one for ‘psychological/counselling’ and another for ‘psychiatric/mhGAP’ practitioners. Each grid features 22–26 competencies, plus optional items for specific interventions. While the structure and content were assessed as logical by supervisors, there were concerns regarding the adequacy of the tool to field reality.

**Conclusions:**

Humanitarian settings have specific needs that require careful consideration when developing capacity-building strategies. Clinical supervision of key competencies through a standardised instrument represents an important step towards ensuring progress of clinical skills among MH practitioners.

## Background

Clinical supervision from a senior mental health (MH) professional imparts knowledge, skills, and competence to novice practitioners (Alfonsson *et al*., 2018; Kühne *et al*., 2019) and helps supervisees manage their own reactions to a patient – especially in conflict, post-conflict and disaster settings, where patients and practitioners are exposed to substantial adversity. A shortage of specialised MH professionals is often the norm in these contexts due to, for example, lack of opportunities for qualification and brain-drain. Professional regulating bodies increasingly require clinicians to achieve competency benchmarks (Falender, 2014; Australian Psychology Accreditation Council, 2019) and with this, a need to define clear accreditation standards has led to the emergence of competency-based supervision approaches, often including competency-based assessment forms (American Psychological Assocation, 2006, p. 273). Competency, here, describes a specific set of knowledge, attitudes and skills, anchored to evidence-based practice and consistent with one's professional qualifications (Barrett *et al*., 2019). It is acquired through regular practice, observation, reflection, corrective feedback and joint planning of specific interventions with the supervisor (Milne *et al*., 2008; Falender and Shafranske, 2017). With increasing experience, professionals are assumed to develop metacompetence, i.e. the ability to reflect on and self-assess their own competency (Falender, 2014; Rønnestad *et al*., 2019) – ‘knowing what you are not able to do’ and recognising personal limits. Supervision practices that positively impact supervisee learning include for example case formulations, supervisor modelling and roleplay, live supervision, and video feedback (Barrett *et al*., 2019). Some clinical supervision models attempt to ensure fidelity to a therapeutic approach or intervention, while others emphasise applying academic knowledge to practice, or learning through reflection.

Competency-based assessment focuses on teachable clinical skills and observable attitudes. The design of assessment approaches, however, requires consensus about which competencies to assess, their expected developmental trajectory, and the behavioural anchors that indicate mastery (American Psychological Association, 2006). There is international consensus on the importance of common factors in therapeutic interventions (e.g. empathy, the therapeutic alliance, social support, adapting to a patient's motivation and resistance) (Roth and Pilling, 2008; Decker *et al*., 2014; Holt *et al*., 2015; Kohrt *et al*., 2015; Elliott *et al*., 2018; Pedersen *et al*., 2020) and often a specific intervention's approach cannot be easily disentangled from these factors. Therefore, clinical supervision must consider characteristics like the intervention's fit to the patient or the in-session rapport established, along with the practitioner's fidelity to a specific technique. To enable learning, effective clinical supervision must also be able to assess specific manual-based as well as abstract, transversal skills (e.g. ‘promoting hope and expectancy of change’) to respond to individual patient needs, promoting therapeutic flexibility over the rigid application of manuals (American Psychological Assocation, 2006; Roth and Pilling, 2008; Faregh *et al*., 2019).

### Supervision of task-shared interventions

In low-resource and humanitarian settings (LRHS), task-sharing care to a less specialised workforce is key to expanding access to services for underserved people with severe and common mental, neurological, and substance use (MNS) conditions (Murray and Jordans, 2016; Singla *et al*., 2017; Patel *et al*., 2018; Carreño *et al*., 2019). In these environments, though supervision models have often been successfully adapted to the needs of generalist health care providers in charge of MNS services, the question of what works and whether and when cultural adaptations are needed remains inconclusive (Cuijpers *et al*., 2018; Heim and Kohrt, 2019). Additionally, successfully adapting supervision models is complicated by the idiosyncratic supervision styles of qualified MH professionals who may change frequently, have different theoretical orientations, and may not be formally trained to supervise others.

Despite the challenges inherent in implementing competency-based supervision in LRHS, some efforts to enhance supervision have occurred in recent years (Kohrt *et al*., 2015). Yet the few tools that exist are limited and lack competency benchmarks to formally identify needs, adapt capacity building strategies, communicate clear expectations, and inform learner self-evaluation. In response to this gap, Médecins Sans Frontières (MSF), a medical humanitarian organization, developed a competency-based supervision tool for use in LRHS (Böhm *et al*., 2022). In MSF settings, mental health activities are set up usually combining some form of psychiatric case management and psychological or psychosocial intervention, typically provided by non-specialists (e.g. doctors, clinical officers, educational psychologists or social support staff who have received initial training in mhGAP approaches). Settings can range from primary health care to hospital-integrated care and may provide support to patients with MNS conditions or patients going through acute and chronic stress (e.g. following injury, forced displacement, chronic illness). Supervision should be provided in person or via telemedicine (joining consultations or discussing cases), preferably by a local expert, or expatriates with experience on a time-limited stay, who in turn have access to telemedicine technical support. The tool is primarily intended for use by those providing and supervising care for severe and common MH conditions, and assesses the applied clinical skills of different care providers, supports their professional development, while attempting to be user-friendly, specific, evidence-informed, multi-disciplinary and applying expertise from multiple specialties. We present here a discussion of the tool's development, providing detail for practitioners who would like to develop similar tools for their context.

## Methods

### Theoretical framework

We employed a qualitative approach of narrative evidence synthesis, rational theoretical scale construction (Simms, 2008) with a co-design method and content analysis. Co-design engages product or service users as part of the development process. Coding was done inductively to generate theory from data (Corbin and Strauss, 2008), which included both pre-existing written material (guidelines, protocols, scales, and tools), and feedback collected in writing and notes. A multi-site approach to tool development was chosen to encompass the diversity of local contexts, project typologies and MH (para)professionals represented in LRHS. While some supervisors may work permanently in one project, others alternate or re-locate over time. This provided a rationale for having a similar approach across places with some flexibility in terms of content. A mixture of purposive sampling (aiming for maximum diversity) and convenience sampling (projects with supervisors willing to take part and time capacity to do so) was used.

### Step 1: identifying core competencies

To design the tool, core competencies, best practice standards for practitioners, and behavioural competency indicators were identified in institutional guidelines, WHO toolkits, and field protocols for MH counselling and psychiatric practices (see online Supplementary Table S1 in appendix). These documents define best practice standards for a competent practitioner, and often provide some behavioural anchors through examples. From the documents, a list of key constructs was created via inductive coding, labelling what the items from different sources intended to measure (e.g. empathy). A literature review was also carried out using PsycINFO and Web of Science with various keyword combinations (‘clinical supervision’, ‘counselling’, ‘psychology’, ‘psychotherapy’, ‘mental health’). Both peer reviewed and grey literature were included, to avoid bias towards content from high income countries. Due to the large number of peer reviewed records on essential elements of counselling or psychotherapy alone (500,000 + ), the search was narrowed down to articles that focused on:

- Mental health care and supervision in LRHS.
- Supervision models and instruments (incl. high income countries) used in practitioner training for specific interventions (e.g. cognitive-behavioural therapy).
- Patient/client-rated experiences of a therapeutic interaction, regardless of the specific intervention delivered (e.g. client perceptions of empathy).

Grey literature documents were included if they met the following selection criteria:

- They were an official institutional guideline, a validated protocol or tool in use in an MSF project. *Or:* They described an instrument used by other organisations, e.g. for clinical supervision of a MH intervention.
- They were available in English or French as of October 2019.
- They focused on individual or group counselling, psychological or psychiatric interventions for common mental health problems and/or more severe conditions – rather than general health education only.
- They focused on clinical supervision, rather than performance management only.

### Step 2: item generation

Using evidence gathered in Step 1, the list of constructs (e.g. ‘rapport’) to be assessed during supervision was generated. Instruments used in prior research were consulted in the development of definitions and descriptors (see online Supplementary Table S1 in appendix). Whenever several descriptors were identified for the same construct in the literature, these different versions were collected to capture all potentially important aspects and maximise representativeness (Simms, 2008). Descriptors gave examples of ‘good practice’ to enable goal setting, and were grouped by intervention (e.g. counselling/psychological, psychiatric/mhGAP, both) and complexity. An easier (Level 1) and more difficult (Level 2) version of each item listed in the tool was developed to match the user's education, professional experience, and proficiency. Finally, all items in the initial pool were coded by the constructs they were assumed to assess. Frequencies of appearance were counted to estimate the weighting given to each construct (without a formal consideration of psychometric properties). Saturation was reached from a pragmatic perspective when a high level of repetition of items was identified.

### Step 3: pilot of the initial item pool

**Fig. 1.**
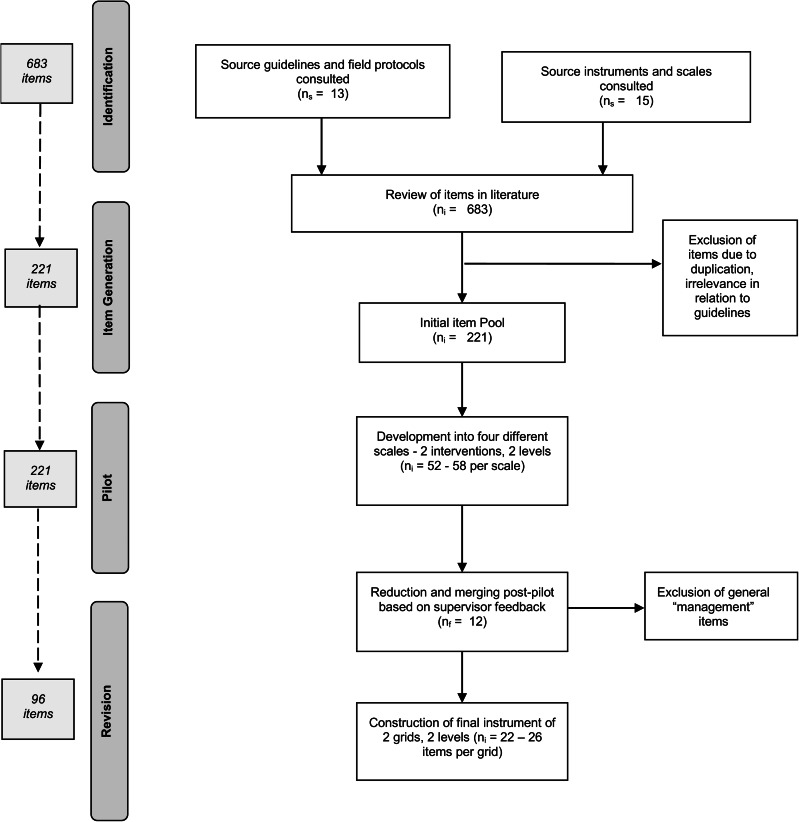
Overview of the skills grid construction process for competency-based supervision (*n*_s_, number of source instruments; *n*_i_, number of items; n_f_, number of feedbacks/grids submitted).

Items in the initial pool (see Fig. 1) were organised into four different skills grids by profession and level. This initial ‘prototype’ tool in Excel, including long descriptors, was then sent to supervisors (psychiatrists, psychologists, and professional counsellors) working in different MSF projects with instructions for a simulation study. Participants were accredited MH professionals (the majority clinical psychologists), with several years of clinical experience, as well as specific current experience in at least one humanitarian project, and a range of experience regarding providing supervision (novice to several years' experience). Participants were asked to select one grid for review based on their own profession and relevance to their current project, and to (1) self-evaluate on each item as a clinician, and (2) imagine giving feedback to another person/supervisee on each item. They were then asked to highlight any items of difficulty, unsuitability or repetition, and comment on them. They were also prompted to suggest alternatives or additional items not already covered.

Twelve supervisors working in seven projects in Liberia, Jordan, Iraq, and the Occupied Palestinian Territories returned a total of 16 completed skills grids (75% return rate). Additional general feedback was given by five more supervisors, who had not had time to complete the task, in one-on-one Skype interviews or via email. Overall, participants had wide international humanitarian experience, including in other countries such as Bangladesh, Central African Republic, Syria, Kenya, Uganda and Malawi.

### Step 4: revision of the grid

A content analysis of qualitative feedback on the returned pilot grids was carried out by the first reviewer with the aim of reducing and refining the items and descriptors. A reduced version of the item pool was then re-coded by constructs and screened by the other two reviewers to merge items and exclude duplicates, with diverging proposals from supervisors discussed between reviewers based on their likely applicability to the largest number of projects. Finally, items were specifically screened for difficult or ambiguous language, since the majority of reviewers and participants were not native English speakers (and technical, abstract language had been common in the descriptors used in academic research). Additional feedback on feasibility of the final grid was obtained through (non-recorded) focus group discussions in two projects, individual interviews via Skype with clinicians in two more, and informal discussion at expatriate briefings and debriefings. Participants were MH practitioners (counsellors and psychologists) in two inpatient hospitals, and were asked by a facilitator to give general feedback on the approach itself, as well as specific feedback on the items, and identify how they thought the tool could be used (or not).

## Results

### Structure of the final skills grid

Two competency tools were created through this development process; one was a skills grid for counsellors or psychologists and the other for mhGAP-trained or psychiatric practitioners.^†^ While the different skills grids are distinct, there is a significant proportion of overlap since they were developed based on the same constructs of good practice (see online Supplementary Table S2 in appendix).

So as to account for the wide variation in skills supervised, two versions of each tool emerged, to be used by different types of supervisors based on the professional role, experience, and educational background of the practitioners. The varied backgrounds and contexts included, for example, clinical officers (a qualification given to nurses or physician assistants who have undergone specific advanced training in clinical psychiatry) providing care for severe and common MH conditions being supervised by a psychiatrist in Liberia, psychosocial counsellors in an Internally Displaced Persons camp in Iraq being supervised by a clinical psychologist, and psychologists providing care for survivors of sexual violence in Kenya. Only two skill levels were finally included for simplicity and usability (beginner/advanced). Per field testing feedback, the Level 1 – beginner tool provides streamlined descriptions of skills. Level 2 – advanced assumes increased meta-competence and helps practitioners adapt standard interventions to the patient and systematically structure treatment plans. Level 2 was found to be best suited to more formally trained MH clinicians (e.g. psychologists or professional counsellors), while other practitioners (e.g. lay counsellors or psychosocial workers) could progress to Level 2 regardless of formal education if they demonstrated mastery of Level 1 skills.

The pilot process reduced the number of items being assessed by half, although merging some items sometimes resulted in longer descriptions (see online Supplementary Table S2 in appendix). The items included in the tool draw from multiple information sources including in-session observation, case formulation, and clinical file review, though evaluating each of these sources will vary depending on the set-up, approach, and resources available. Supervision using the tool is carried out in a supervisee-focused style since the items focus on practitioner behaviours, although they refer to what the supervisee is doing *in relation to* the patient's needs and prompt discussion about the therapeutic process (client focus).

The tool's final individual item rating scale was streamlined to three points (‘needs a lot of supervision’, ‘needs some supervision’, ‘model for others’) based on supervisor and supervisee feedback. No numerical scoring system was implemented, with the rating scale simplified and focusing on the support needed (rather than a supervisee's ability) in order to promote a qualitative discussion and professional development. Comment boxes allow supervisors and supervisees to qualify their rating and detail support plans for future review.

### Content of the final skills grid

**Fig. 2.**
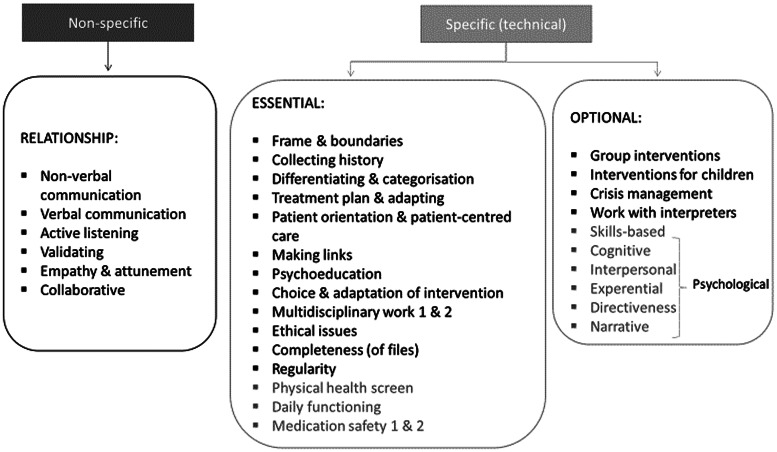
Items of the different skills grids. Items in black exist across all grids. Items in dark grey are not included across all four grids.

The toolkit was designed to contain behavioural descriptions and examples of ‘good practice’ as a basis for self-reflection, objective and specific observer evaluation, and discussion. It covers common factors of therapeutic care as well as technical aspects of how therapeutic interventions are delivered (see Fig. 2). For example, psychoeducation is a universal feature of mental health care to destigmatise and empower patients understand and manage their condition, but the expectation (e.g. to communicate a diagnosis or not) is different by profession. For the non-specific, common factor items, there was no distinction between mhGAP-trained/psychiatric and counselling/psychological practitioners and little or no progression between Level 1 and Level 2. For the specific items, the content differed in terms of activity (counselling or mhGAP) and level of difficulty and progression (see e.g. item 15 on assessment in online Supplementary Table S2).

Because of conflicting feedback during the pilot testing phase of development, more specific examples were kept on common factors of interventions (e.g. recovery goal setting, multidisciplinary work), with optional items for intervention content (e.g. groups). Participants also gave relevant qualitative feedback on common problems in projects, such as challenges in multidisciplinary teamwork, or concerns about language (incomprehensibility, misunderstandings) or bias introduced by inexperienced supervisors with limited knowledge of the local culture and context. This ‘added feedback’ informed the additional materials for supervisors in the final toolkit.

## Discussion

This article set out to the describe the process and result of a competency-based supervision tool development and pilot in multiple LRHS. The final tool presented and freely available draws on existing competency-based training models in professional psychology and the competency grids developed by academic institutions (see online Supplementary Table S1 in appendix) as well as mh-GAP practice elements, but uses simplified language and concrete examples as models for practice. It is not an assessment tool in and of itself but relies on supervisors with professional MH experience being prompted by the tool to give real examples and relate standards of practice to what they are observing. It also provides a way for practitioners to monitor their own development and bring up questions. It should be noted that the pilot tool developed in 2019 prioritises common factors of treatment emphasised in task-sharing intervention manuals (Pedersen *et al*., 2020), although some of the terminology is specific (e.g. ‘validating’ as a form of active listening).

### Implementation feedback

Supervisor feedback on the utility of the tool overall was positive. Suggested frequency of use ranged from day-to-day practice (as a tool for live observation and discussion after session) to every three months (as a tool for formal evaluation). There were, however, concerns about a potentially ‘restrictive’ effect on clinical supervision in terms of content (leaving little room for flexibility in what to discuss), and its usability in day-to-day practice with already limited time for supervision.

Another concern was confidentiality of clinical supervision and its relationship with performance management. Having a numerical ‘score’ was perceived as potentially useful to assess and document progress in more objective terms, which could inform human resources decision-making. However, this was firmly rejected by other supervisors, who were concerned about a negative effect on supervision quality by overly focusing on a perfect score, which could trigger competition between practitioners and jeopardise the original purpose of identifying and mobilising the resources necessary to support professional development and enhance overall quality of care. In considering implementation, supervisors highlighted the need for transparency about the use of tools. No scores were included in the final version, which has been used in training supervisors, since other evaluation tools and pathways exist for the purposes of performance management.

### Limitations

This was an implementation trial driven by a need to systematise supervision practices for diverse projects in the humanitarian context. As such, the approach applied prioritised the development of a ‘usable’ end-product over the development of a scale with psychometric properties. Items were gathered in Step 3 based on qualitative feedback from supervisors with varying levels of practical field experience, not a Delphi process. Supervisor appraisal of a given item may or may not reflect the actual importance of the item for ‘good supervision’. While instruments constructed in a purely rational theoretical framework can achieve good convergent validity, their discriminant validity is often poor (Simms, 2008). For example, the skills grid may conflate supervisee skills with supervisor/supervisee rapport or other institutional factors. The assumption that competency development is linear and requires continuous direct evaluation and feedback is also based on western academic models of teaching medicine and psychology, and may not be the preferred way of teaching or learning in other contexts.

The post-pilot scale of 22 to 26 items plus optional items is still quite comprehensive, and supervisees or supervisors will not be able to hold in mind this number of items. Some items can be assumed to be more critical to provision of care than others, but we lack the evidence on the relative contribution of each to patients' outcomes. This challenge has been identified in other competency frameworks (Roth and Pilling, 2008; Kohrt *et al*., 2015). A prospective review of the tool therefore invited supervisors and practitioners explicitly to identify parts of the tools they found not useful for their setting and/or repetitive.

In order to ensure that clinical supervision remains a ‘safe base’ for learning that provides room for corrective experiences (Borders *et al*., 2014; Rønnestad *et al*., 2019), supervisors and supervisees are prompted to identify and discuss no more than two or three points in depth, based on what they are observing in consultations and/or case discussions. In a real-life setting, implementation will need to be driven by the specific situation, e.g., live shadowing ‘basic counselling’ in an overwhelmed health care structure, remote supervision in high security contexts, or telemedicine case discussion for severe psychiatric presentations. This need for individual tailoring means dependence on the presence of experienced supervisors.

Furthermore, the *supportive* (restorative) aspect of clinical supervision is only addressed implicitly in the toolkit through additional introduction and training materials. Aspects of intercultural dynamics, power and privilege in humanitarian settings are likewise not addressed explicitly, although they have been critically discussed as important in task-sharing (Kemp *et al*., 2019). While psychotherapeutic clinical supervision literature has increasingly begun to discuss both of these issues (Ivers *et al*., 2017; Lee, 2018; Watkins *et al*., 2019), there is a shortage of practical recommendations on what this means both in MH interventions and in medicine more broadly (MacDonald and Ellis, 2012) and this certainly requires addressing. While treatment manuals often rest on evidence from quasi-experimental designs or randomised control trials, clinical supervision models rely more heavily on assumptions and supervisee feedback (Simpson-Southward *et al*., 2017; Kühne *et al*., 2019).

### Post-pilot implementation

The pilot tool was developed in late 2019. 2020 and 2021 were marked by the COVID-19 pandemic, a loss of access to populations, significant interruption of medical activities, and competing humanitarian priorities, which initially hindered systematic implementation of the toolkit across contexts. At the same time, many shifts have occurred globally affecting the nature and modality of clinical supervision. There has been an increasing focus on its supportive role, on the necessity of providing learning opportunities in task-shifted interventions, and innovation in the areas of telemedicine, remote supervision and app-based learning. Furthermore, a more recent systematic review highlighted the importance of common factors across different treatment manuals commonly employed in task-sharing (Pedersen *et al*., 2020), while a qualitative study with supervisors highlighted similar challenges to ours in balancing managerial, ‘objective-focused’ and supportive aspects of supervision in humanitarian emergencies (Perera *et al*., 2021). Compared to 2019, the focus now has shifted more towards MH practices in LRHS, rather than ‘adapting approaches from the West’, as was done in some ways by initially reviewing competency tools from high income contexts. At the same time, health care resources globally have been impacted by the primary and secondary effects of the pandemic. In LRHS in particular, this means that there are a multitude of competing demands on health care workers, and that providing some form of MH care may be mistakenly considered an ‘add on’ responsibility.

MSF's experience so far has been that there is often a demand for tools as a starting point, although their use may be less systematic in practice. In the contexts where the competency-based supervision tool has been used, it has been either modified (shortened/simplified) or used ad hoc for a specific purpose, with learning goals formulated based on it. For example, a large MNS project with frequent psychiatric supervisor gaps used it to assess the ‘status quo’, to review the impact of all trainings and supervision historically provided by MSF and other organisations, then provide refresher trainings with practical roleplays based on common needs identified across the team, and subsequently developed support plans for individual staff, which have then been systematically reviewed every three months. The tool is also envisioned as a useful basis for remote supervision in contexts where access is an issue, or to continue remote discussions after initial face-to-face supervision and seeing patients together. It has been found less useful for projects with only a very small psychosocial component (no psychiatric/psychological care) that do not use a ‘clinical’ frame. A two-year test of the ‘final’ version of the tool, with or without the supporting materials, was agreed in November 2021. This test also aims to assess how useful the tool is to supervisees, if and how supervisors take learnings from one context to another, and whether different LRHS could interconnect, for example, through shared discussions.

### Recommendations for future research

In order to evaluate the four skills grids constructed, a simulation study in the context of training could be envisioned with the explicit aim of item reduction to increase usability, or to compare with other measures – for example, by using roleplays (Kohrt *et al*., 2015). Given that other projects (e.g. EQUIP) are developing tools for psychological interventions, this exercise may not be a justifiable investment of resources. However, a supervision simulation trial relevant for mhGAP-trained non-specialist clinicians is certainly identified as a gap.

Outside the scope of this particular instrument, there are still some knowledge gaps regarding clinical supervision. Despite existing evidence that it facilitates supervisee skills development (Wheeler and Richards, 2007; Rakovshik *et al*., 2016; Kühne *et al*., 2019), systematic reviews have called for further research with increased methodological rigour (Allan *et al*., 2017; Kühne *et al*., 2019). The evidence that supervision affects patient outcomes is even weaker and inconclusive (Watkins, 2011; Simpson-Southward *et al*., 2017; Alfonsson *et al*., 2018). Only some small, older studies showed higher retention in care, greater symptom reduction and higher patient satisfaction in supervised, specific interventions compared to non-supervised ones (Bambling *et al*., 2006; Bradshaw *et al*., 2007). Nonetheless, supervision remains a key teaching practice to professional competency development, and the most direct form of assuring quality of care.

The dearth of evidence on patient outcomes is even more obvious in humanitarian MH interventions, beyond descriptive analyses of program outcome data. Challenges to clinical supervision such as supervisors' high turn-over and theoretical diversity need to gain centrality when studying and adapting approaches to these settings. An effort was made to include supervisors with different professional backgrounds working in diverse projects in the construction of this tool, to avoid overemphasis of one treatment philosophy or a particular project specialisation. An important next step will be involvement of practitioners in improving the grid and obtaining feedback on supervision practices more broadly from their perspective. The review of supervision and clinical notes could provide further insight into reflective processes, since counselling and psychotherapeutic interventions essentially deal with change processes (Rodgers and Elliott, 2015). Finally, the involvement of people with lived experience (service users/patients) in research could provide a better understanding of what we mean by ‘outcome’: for example, how they experience the therapeutic interaction and the presence of an ‘extra person’ in the case of live supervision.

## Conclusions

This paper set out to describe the process of constructing an evidence-informed, standardised tool for use in mental health activities in humanitarian contexts. Rather than presenting a perfect end product, it outlines the development of tools adapted to specific learning conditions and clinical environments of humanitarian settings. It is also based on a small pilot, and it is assumed that the tool will require adaptation for different professional, cultural and linguistic contexts. We therefore actively invite feedback, and hope to continue the discussion on providing evidence-based care in LRHS. While theoretical approaches and research evidence from different settings can inform each other, contextually relevant evidence can only grow from practice and systematic feedback in the actual setting itself.

## Footnotes

† The notes appear after the main text.

## Supplementary material

For supplementary material accompanying this paper visit http://dx.doi.org/10.1017/gmh.2022.53. click here to view supplementary material
