# Supplementary material for: What matters in mental health care? A co-design approach to developing clinical supervision tools for practitioner competency development
Source: Glob Ment Health (Camb). 2022 Oct 21;9:491–8. doi: 10.1017/gmh.2022.53 (PMC9807002; doi:10.1017/gmh.2022.53)
Supplement: Supplementary file 1 [file S205442512200053Xsup.zip › S205442512200053Xsup001.docx]

## Table 1. Instruments consulted for generation of the initial supervision items and external and internal (Médecins sans Frontières; MSF) sources.

| **Instrument** | **Source** | **Target Group** | **Location** | **# of Items** |
| --- | --- | --- | --- | --- |
| Core competencies for MNS | Collins et al., 2015 | Non-specialists treating MNS in sub-Sahara Africa. Developed in Uganda | Uganda | 24 |
| Mental health gap (mhGAP) core competencies | World Health Organization, 2016a | Non-specialist health care workers receiving clinical supervision post-mhGAP training |  | 45 |
| Enhancing Assessment of Common Therapeutic factors (ENACT) | Kohrt et al., 2015 | mhGAP-trained, non-specialist health care workers providing MNS care | Nepal, Liberia, Uganda | 18 |
| Accreditation Standards | Australian Psychology Accreditation Council, 2019 | Psychology graduates (Masters level). Excluded “specific areas of practice”. |  | 17 |
| Clinical Psychology Practicum Competencies Rating Scale (CYPRS) | Gonsalvez et al., 2015 | Clinical psychology trainees in Australia, internship evaluation. | Australia | 69 |
| Revised competency benchmarks in professional psychology | American Psychological Association, 2012 | Psychologists at three different stages of professional development in the U.S. (practicum, internship, professional practice). | US | 55 |
| Competencies for psychology practice in primary care | American Psychological Association, 2015 | Psychologists practicing in primary care settings in the U.S. | US | 85 |
| Supervisor Trainee Quarterly Evaluation (STQE) | Callahan et al., 2017 | Trainee psychologists (doctoral students) in internship in the U.S. | US | 25 |
| Therapist Empathy Scale (TES) | Decker et al., 2014 | Therapist empathy scale in the U.S. developed with practicing clinicians | US | 9 |
| Centre for Outcomes Research and Effectiveness (CORE )competence frameworks | University College London, 2016 | MH clinicians delivering specific therapeutic interventions in various settings Disorder-specific intervention items were excluded | UK | 169 |
|  |  | _Cognitive-behavioural therapy (CBT) map_ |  | _43_ |
|  |  | _Child and adolescent mental health services (CAMH)s map_ |  | _32_ |
|  |  | _Interpersonal psychotherapy map_ |  | _34_ |
|  |  | _Psychoanalytic/psychodynamic map_ |  | _30_ |
|  |  | _Interventions for persistent physical problems map_ |  | _30_ |
| Problem Management Plus (PM+) | World Health Organization, 2016b | Non-specialist helpers delivering the PM+ intervention. |  | 35 |
| Checklist for roleplays | Viciana, 2009 | Counsellors delivering individual, basic counselling within humanitarian NGO |  | 37 |
| Logbook community health educator | MSF | Developed for performance evaluation of community health educators | Various | 18 |
| Counselor competency checklist | MSF | Developed for psychosocial counselor training and supervision | Iraq | 38 |
| MH clinician performance appraisal form | MSF | Developed for staff evaluation of prescribing clinicians, training development | Liberia | 39 |

**References**

**American Psychological Association** (2012) *Revised Competency Benchmarks in Professional Psychology* [Online]. Available at https://​www.apa.org​/​ed/​graduate/​benchmarks-​evaluation-​system (Accessed 27 January 2020).

**American Psychological Association** (2015) *Competencies for Psychology Practice in Primary Care* [Online]. Available at http://​www.apa.org​/​ed/​resources/​competencies-​practice.pdf (Accessed 2 January 2019).

**Australian Psychology Accreditation Council** (2019) *Accreditation Standards for Psychology Programs* [Online], Sydney. Available at https://​www.psychologycouncil.org.au​/​standards_​review (Accessed 28 January 2020).

**Callahan, J. L., Neumann, C. S., Cox, R. J. and Ruggero, C. J.** (2017) ‘The Supervisor Trainee Quarterly Evaluation (STQE): Psychometric support for use as a measure of competency’, *Training and Education in Professional Psychology*, vol. 11, no. 3, pp. 117–127.

**Collins, P. Y., Musisi, S., Frehywot, S. and Patel, V.** (2015) ‘The core competencies for mental, neurological, and substance use disorder care in sub-Saharan Africa’, *Global Health Action*, vol. 8.

**Decker, S. E., Nich, C., Carroll, K. M. and Martino, S.** (2014) ‘Development of the Therapist Empathy Scale’, *Behavioural and cognitive psychotherapy*, vol. 42, no. 3, pp. 339–354.

**Gonsalvez, C. J., Deane, F. P., Blackman, R., Matthias, M., Knight, R., Nasstasia, Y., Shires, A., Nicholson Perry, K., Allan, C. and Bliokas, V.** (2015) ‘The Hierarchical Clustering of Clinical Psychology Practicum Competencies: A Multisite Study of Supervisor Ratings’, *Clinical Psychology: Science and Practice*, vol. 22, no. 4, pp. 390–403.

**Kohrt, B. A., Jordans, M. J. D., Rai, S., Shrestha, P., Luitel, N. P., Ramaiya, M. K., Singla, D. R. and Patel, V.** (2015) ‘Therapist competence in global mental health: Development of the Enhancing Assessment of Common Therapeutic factors (ENACT) rating scale’, *Behaviour research and therapy*, vol. 69, pp. 11–21.

**University College London** (2016) *UCL competence frameworks for the delivery of effective psychological interventions* [Online], London, UCL. Available at https://​www.ucl.ac.uk​/​pals/​research/​clinical-​educational-​and-​health-​psychology/​research-​groups/​core/​competence-​frameworks (Accessed 2 January 2019).

**Viciana, K. M.** (2009) *Supervision and Training On-the-Job Guide for MSF Psychosocial Programmes,* MSF - Operational Centre Brussels.

**World Health Organization** (2016a) *mhGAP Training of Health-care Providers (ToHP) training manual: version 2.0 for field testing* [Online]. Available at https://​www.who.int​/​mental_​health/​mhgap/​training_​manuals/​en/​ (Accessed 27 January 2020).

**World Health Organization** (2016b) *Problem Management Plus (PM+): Individual psychological help for adults impaired by distress in communities exposed to adversity* [Online]. Available at https://​www.who.int​/​mental_​health/​emergencies/​problem_​management_​plus/​en/​ (Accessed 27 January 2020).
