# Supplementary material for: What matters in mental health care? A co-design approach to developing clinical supervision tools for practitioner competency development
Source: Glob Ment Health (Camb). 2022 Oct 21;9:491–8. doi: 10.1017/gmh.2022.53 (PMC9807002; doi:10.1017/gmh.2022.53)
Supplement: Supplementary file 1 [file S205442512200053Xsup.zip › S205442512200053Xsup002.docx]

**Table 2**. *Overview of post-pilot items and weighting (October 2019). ‘Counselling/psychological’ can be used in working with counsellors or psychologists depending on level of experience (professional education and training, clinical experience), whereas ‘mhGAP/psychiatric’ can be used for mhGAP-trained health care workers or clinical officers, nurses and doctors providing psychiatric care. No. in first column reflects the relative position in each supervision grid (Counselling L. 1/Counselling L. 2/ …).*

| **No.** | **Assumed Constructs** | **Counselling/psychological – LEVEL 1** | **Counselling/psychological –**  **LEVEL 2** | **mhGAP/psychiatric – Level 1** | **mhGAP/psychiatric - LEVEL 2** |
| --- | --- | --- | --- | --- | --- |
| **A. Observed Transversal Skills** | | | | | |
| *1. Communication with the patient (live)* | | | | | |
| 1. | Responsive-ness/  Sensitivity/  Communica-tion | **Non-verbal communication**: Body language is appropriate and helps the patient feel comfortable (e.g. open posture). Professional stays focused, does not appear "distracted". | **Non-verbal communication:** Body language is appropriate and helps the patient feel comfortable (e.g. open posture). Professional stays focused, does not appear "distracted". | **Non-verbal communication:** Body language is appropriate and helps the patient feel comfortable (e.g. open posture). Professional stays focused, does not appear "distracted". | **Non-verbal communication:** Body language is appropriate and helps the patient feel comfortable (e.g. open posture). Professional stays focused, does not appear "distracted". |
| 2. | Responsive-ness/  Communicat-ion | **Verbal communication:** Language is clear and adapted to the patient. Professional changes language when the patient does not understand, checks understanding. Speed is appropriate. Pauses. | **Verbal communication:** Language is clear and adapted to the patient. Professional changes language when the patient does not understand, checks understanding. Speed is appropriate. Pauses. | **Verbal communication:** Language is clear and adapted to the patient. Professional changes language when the patient does not understand, checks understanding. Speed is appropriate. Pauses. | **Verbal communication:** Language is clear and adapted to the patient. Professional changes language when the patient does not understand, checks understanding. Speed is appropriate. Pauses. |
| 3. | Rapport | **Active listening:** Professional paraphrases, summarises, clarifies, shows understanding. | **Active listening:** Professional paraphrases, summarises, clarifies, shows understanding. | **Active listening:** Professional paraphrases, summarises, clarifies, shows understanding. | **Active listening:** Professional paraphrases, summarises, clarifies, shows understanding. |
| 4. | Empathy | **Empathy:** Professional tries to give words to the patient's feelings in a gentle way. Tone is kind, calm and communicates focused interest. | **Empathy:** Professional responds warmly and calmly to patient, is attuned to the patient in session and can modulate own emotional response appropriately (being either at, slightly above to below patient's level of arousal, as appropriate). | **Empathy:** Professional tries to give words to the patient's feelings in a gentle way. Tone is kind, calm and communicates focused interest. | **Empathy:** Professional responds warmly and calmly to patient, is attuned to the patient in session and can modulate own emotional response appropriately (being either at, slightly above to below patient's level of arousal, as appropriate). |
| 5. | Empathy | **Non-judgmental:** Comments to the patient and reflections of their situations are stated in a matter-of-fact manner, without casting judgment or blaming the patient. | **Non-judgmental:** Comments to the patient and reflections of their situations are stated in a matter-of-fact manner, without casting judgment or blaming the patient. | **Non-judgmental:** Comments to the patient and reflections of their situations are stated in a matter-of-fact manner, without casting judgment or blaming the patient. | **Non-judgmental:** Comments to the patient and reflections of their situations are stated in a matter-of-fact manner, without casting judgment or blaming the patient. |
| 6. | Empathy/  Communica-tion | **Validating**: Normalising the patient’s reactions or letting the patient know their emotional reactions are understandable. Professional does not minimise the feelings or tell the patient to feel differently. | **Validating:** Normalising the patient’s reactions or letting the patient know their emotional reactions are understandable. Professional does not minimise the feelings or tell the patient to feel differently. | **Validating:** Normalising the patient’s reactions or letting the patient know their emotional reactions are understandable. Professional does not minimise the feelings or tell the patient to feel differently. | **Validating:** Normalising the patient’s reactions or letting the patient know their emotional reactions are understandable. Professional does not minimise the feelings or tell the patient to feel differently. |
| 7. | Rapport/  Sensitivity/  Communica-tion | **Feedback:** Notices when the patient is sharing difficult topics and praises their openness and sharing. Gives positive and supportive feedback to the patient, encourages progress. Reacts to feedback from patient supportively. | **Feedback:** Notices when the patient is sharing difficult topics and praises their openness and sharing. Gives positive and supportive feedback to the patient, encourages progress. Reacts to feedback from patient supportively. | **Feedback:** Notices when the patient is sharing difficult topics and praises their openness and sharing. Gives positive and supportive feedback to the patient, encourages progress. Reacts to feedback from patient supportively. | **Feedback:** Notices when the patient is sharing difficult topics and praises their openness and sharing. Gives positive and supportive feedback to the patient, encourages progress. Reacts to feedback from patient supportively. |
| 8. | Rapport | **Collaborative:** Professional encourages the patient to take an active part in the session. They do not inappropriately tell the patient what to do in a situation (give advice) but encourage thinking. | **Collaborative:** Professional encourages the patient to take an active part in the session. They do not inappropriately tell the patient what to do in a situation (give advice) but encourage thinking. | **Collaborative:** Professional encourages the patient to take an active part in the session. They do not inappropriately tell the patient what to do in a situation (give advice) but encourage thinking. | **Collaborative:** Professional encourages the patient to take an active part in the session. They do not inappropriately tell the patient what to do in a situation (give advice) but encourage thinking. |
| 9. | Empathy | **Communicating concern**: Professional reviews file before session to know what was discussed before. Remembers (in session) what was discussed before and makes links for the patient. | **Communicating concern**: Professional reviews file before session to know what was discussed before. Remembers (in-session) what was discussed before and makes links for the patient. | **Communicating concern**: Professional reviews file before session to know what was discussed before. Remembers (in-session) what was discussed before and makes links for the patient. | **Communicating concern**: Professional reviews file before session to know what was discussed before. Remembers (in-session) what was discussed before and makes links for the patient. |
| *2. Organisation of session (live)* | | | | | |
| 10. | Organisation/  Responsive-ness | The room and the material needed is ready (toys, tools, paper, scales used, …). | The room and the material needed is ready (toys, tools, paper, scales used, …) and appropriate to the developmental and stimulation needs of the patient (not too much, not too little). | The day-to-day pharmacy stock is stored and maintained appropriately, and available. | The day-to-day pharmacy stock is stored and maintained appropriately, and available. |
| 11. | Organisation/Boundaries | Sessions begin and are ended in a timely manner. | Sessions begin and are ended in a timely manner, and follow a structure adapted to the capacity of the patient to engage. | Consultations begin and are ended in a timely manner. | Consultations begin and are ended in a timely manner, and follow the capacity of the patient to engage. |
| 12. | Ethics/  Boundaries | Confidentiality is maintained; the professional does not discuss the patient’s concerns in front of other people. | Confidentiality is maintained; the professional does not discuss the patient’s concerns in front of other people. | Confidentiality is maintained; the professional does not discuss the patient’s concerns in front of other people. | Confidentiality is maintained; the professional does not discuss the patient’s concerns in front of other people. |
| 13. | Organisation/continuity | Professional schedules the next follow-up session. | Professional schedules the next follow-up session. | Professional schedules the next follow-up session. | Professional schedules the next follow-up session. |
| \| **B. Observed Technical Skills** \| \| --- \| | | | | | |
| *1. In session (live)* | | | | | |
| 14. | Assessment/  structure | Professional explains frame of intervention, own role/limits and confidentiality clearly. | Professional explains frame and purpose of psychological intervention and confidentiality clearly, obtains informed consent. | Professional explains frame and purpose of consultation clearly, obtains informed consent. | Professional explains frame and purpose of prescriber intervention clearly, obtains informed consent. |
| 15. | Assessment/  structure | Professional conducts a semistructured assessment, using the main tools available and following the main concerns of the patient. | Professional conducts a semistructured assessment based on psychological interviewing techniques, using standardised tests where appopriate/available. | Professional conducts a diagnostic interview, including taking a history of the main complaint and medical history. | Professional conducts a structured or semi-structured diagnostic interview, including comprehensive history taking and assessment of mental status. |
| 16. | Assessment/  Differentia-tion/  sensitivity | Professional supports the patient in expressing emotions and is able to listen to painful feelings. | Professional supports the patient in expressing emotions and clarifying own difficulty, can express nuances in emotions and ambivalences for the patient and reflect on them. | Professional asks questions or performs examinations related to screening for common conditions not disclosed (e.g. substance use, infection, …). | Professional asks questions or performs examinations related to screening for common conditions not disclosed (e.g. substance use, infection, …). |
| 17. | Assessment/  sensitivity/  risk | Professional follows up on "hints" of patient, is able to gently ask questions about difficult subjects (e.g. suicidal thoughts, experiences of violence). | Professional systematically assesses risk factors such as suicidal ideation or protection concerns in a graded manner, adapted to the patient’s level of disclosure. | Professional follows up on "hints" of patient, is able to gently ask questions about difficult subjects (e.g. suicidal thoughts, experiences of violence). | Professional systematically assesses risk factors such as suicidal ideation or thoughts of harming others and is able to respond supportively. |
| 18. | Assessment/  crisis/  risk/trauma | Professional is able to identify signs of an acute crisis (e.g. patient becomes very distressed, talks about wanting to end his/her life) and refers for more specialised support. | Professional is able to stabilise a patient in an acute crisis (e.g. acutely suicidal, panic attacks, dissociation), with the appropriate level of support and behavioural techniques. | Professional is able to identify signs of an acute crisis (e.g. patient is incoherent) and seeks specialised advice where needed. | Professional is able to stabilise a patient in an acute crisis (e.g. acutely suicidal, catatonia, distressing positive symptoms of psychosis, …), with the appropriate level of pharmacological intervention. |
| 19. | Treatment/  Responsive-ness/  Directive-ness | Professional supports the patient in considering a different perspective on a problem and can point out different sides of an issue. | Professional adapts own directiveness to readiness and level of resistance encountered by the patient (e.g. integrates motivational interviewing approach where required). | Professional adapts medical information to the patient’s level of understanding, concentration etc. | Professional adapts own directiveness to engagement of the patient in providing psychoeducation and follow-up. |
| 20. | Treatment/  Theoretical knowledge/  therapeutic technique | Professional gives accurate psychoeducation (information) on symptoms experienced, using specific examples. | Professional gives accurate psychoeducation (information) on mental health conditions, specific to the patient and using examples, picking up on comparisons/images used by the patient as needed. | Professional gives accurate psychoeducation (information) on symptoms experienced, using specific examples. | Professional gives accurate biopsychosocial psychoeducation on mental health condition experienced, taking into account the patient's health knowledge and beliefs. |
| 21. | Treatment/  recovery orientation/  therapeutic techniques | Professional selects exercises/interventions that support the patient in coping with his/her symptoms (e.g. problem-solving, breathing exercises) and demonstrates them. | Professional uses insight-oriented or symptom reduction/behaviour change techniques according to indication, with a clear rationale for the patient. | Professional asks about and supports functioning in daily activities. | Professional supports behavioural health strategies for managing symptoms and promotes functioning in daily activities. |
| 22. | Treatment/  structure/  therapeutic techniques | Professional is specific in planning with the patient, breaks large goals down into smaller steps and takes the patient’s ability into account. | Professional structures and prioritises the treatment plan with the patient based on evidence (e.g. stabilisation and restoring a sense of control first). | Professional agrees goals for the treatment with the patient, and explains the next steps. | Professional structures and prioritises the treatment plan with the patient based on evidence (e.g. stabilisation and restoring a sense of control first). |
| 23. | Treatment/  Responsive-ness/thera-peutic techniques | Professional is able to tolerate and contain moments of conflict expressed by the patient, and reacts in a non-confrontational manner. | Professional attends to moments of inter- or intrapersonal conflict with the patient, is able to explore them with the patient and responds in a way that allows for a different relationship experience. | Professional checks for barriers to adherence, and uses a problem-solving approach for addressing them with the patient/caregiver. | Professional assesses barriers to adherence in a non-confrontational manner and works with the patient/caregiver on problem-solving. |
| 24. | Treatment/  rapport/reco-very orientation | Professional concludes the counselling process with a patient appropriately, reviewing the follow-up and supporting the patient’s goals for the future. | Professional concludes the therapeutic process with a patient appropriately and supportively, makes a relapse prevention plan and instils hope that recovery is possible. | Professional concludes the treatment with a patient appropriately and supportively, tapering medication gradually. | Professional concludes the treatment with a patient appropriately and supportively, makes a relapse prevention plan and tapers medication gradually. |
| 25. | Treatment/  responsive-ness/  therapeutic techniques | Professional uses expressive techniques with children , e.g. by integrating play and art activities to understand the child’s inner world. | Professional appropriately uses expressive techniques with children , e.g. by integrating play and art activities into the treatment with a specific rationale. | Professional assesses children with adapted language, and carefully considers whether there is a need for a pharmacological intervention. | Professional assesses children with adapted language, and carefully considers whether there is a need for a pharmacological intervention. |
| 26 . | Treatment/  therapeutic techniques | Professional involves caregivers in counselling children, and works with the family on restoring routines and a secure environment. | Professional systematically includes caregivers in the treatment of children, providing appropriate parenting guidance and a therapeutic understanding the child’s difficulties within the context of the family system. | Professional systematically includes caregivers in the treatment of children, providing basic information about child development. | Professional systematically includes caregivers in the treatment of children, providing psychoeducation about child development and support for psychosocial interventions. |
| 27. | Treatment/  boundaries | Professional maintains a professional relationship with patients, distinguishes this from friendship (e.g. does not have private contact). | Professional maintains a professional relationship with patients, is able to discuss wishes and boundary issues with the patient where the patient makes a request the Professional cannot fulfil. |  |  |
| 28. | Treatment/  therapeutic techniques/  trauma | Professional supports the patient in understanding what is happening to them and relating it to before/after, rather than imposing their own explanation. | Professional supports the patient in regaining meaning and integrating experiences into their life history, rather than imposing their own interpretation. |  |  |
| 29. | Treatment/  structure/  therapeutic techniques/  directive-ness | Professional provides a safe frame in the session, summarises and closes session appropriately with a warning before. | Professional provides a safe frame in the session, summarises and closes session appropriately, gives and reviews homework where appropriate. |  |  |
| 30. | Treatment/  recovery orientation/  therapeutic techniques |  | Professional engages the patient as an active participant and supports the patient in gradually tolerating affect, experiencing mastery and regulating behaviours. |  |  |
| 31. | Treatment/  therapeutic techniques |  | Professional supports the patient in exploring and differentiating thoughts, feelings, wishes, beliefs and assumptions in relation to their reality. |  |  |
| 32. | Treatment  /therapeutic techniques/  directive-ness |  | Professional integrates experiential techniques and skills training such as psychodrama, roleplays, schema therapeutic elements etc. where appropriate. |  |  |
| 33. | Treatment/  therapeutic techniques/directive-ness/groups |  | Professional structures group sessions, engaging participants in a process of modelling for and supporting each other and eliciting their feedback, rather than lecturing the group. |  |  |
| 27./  27. | Treatment/  medical |  |  | Professional checks the physical condition of the patient, noting weight, age, vital signs as appropriate. | Professional checks the physical condition of the patient, noting weight, age, vital signs as appropriate. |
| 28./  28. | Treatment/  medical/  psychoedu-cation |  |  | Professional educates patient (and, where appropriate, caregiver) about risks and benefits of treatment, potential side-effects, duration of treatment, and importance of adherence. | Professional educates patient (and, where appropriate, caregiver) about risks and benefits of treatment, potential side-effects, duration of treatment, and importance of adherence. |
| 29./  29. | Treatment/  medical/risk/theoretical knowledge |  |  | Professional checks for contra-indications before prescribing any medication. | Professional checks for contra-indications before prescribing any medication. |
| 30./  30. | Treatment/  medical/risk/theoretical knowledge |  |  | Professional monitors changes in condition and side effects of medication carefully and takes appropriate action. | Professional monitors changes in condition and side effects of medication carefully and takes appropriate action. |
| 31. | Treatment/  medical/risk/theoretical knowledge |  |  |  | Professional provides follow-up according to MSF protocols, with higher frequency in the beginning, increasing dosages gradually and avoiding polypharmacy where possible. |
| *2. In case discussion (non-live)* | | | | | |
| 30./  33./  31./  32. | Diagnosis/  Differentia-tion | Professional identifies the "main complaint" and problem of the person at this moment and chooses a syndrome. | Professional provides an appropriate diagnosis based on differentiated assessment and history taking. | Professional identifies the "main complaint" and problem of the person at this moment and chooses a syndrome. | Professional provides an appropriate diagnosis based on differentiated assessment and history taking. |
| 31./  34./  32./  33. | Assesssment/  Differentia-tion/diagnosis | Professional is specific about the difficulties of a patient, rather than generalising or using an explanation based on “all patients with this problem”. | Professional uses a holistic approach to understanding the difficulties of the patient: integrating thoughts, feelings, behaviours, interpersonal relationships, learning history, environmental and physical factors. | Professional is specific about the difficulties of a patient, rather than generalising or using an explanation based on “all patients with this problem”. | Professional uses a biopsychosocial approach to understanding the difficulties of the patient: integrating psychosocial and environmental factors into the biomedical explanation of their difficulties. |
| 32./  35./  33./  34. | Treatment/  resource orientation | Professional can discuss the patient as a "whole person", rather than focusing on the symptoms alone. | Professional can discuss the patient as a "whole person" and consider different domains of functioning, as well as the patient’s capacity to reflect on their situation. | Professional can discuss the patient as a "whole person", rather than focusing on the symptoms alone. | Professional can discuss the patient as a "whole person" and consider different domains of functioning, rather than using a mechanical explanation. |
| 33./  36./  34./  35. | Assessment/  observation | Professional can discuss observations of the patient, focusing not only what the patient is "saying". | Professional can discuss clinical observations of the patient’s presentation and interaction, the match between verbal and non-verbal behaviour, and take this into account in the treatment plan. | Professional can discuss observations of the patient, focusing not only what the patient is "saying". | Professional can discuss clinical observations of the patient and mental status in a structured way. |
| 34./  37./  35./  36. | Treatment/  hypotheses/  responsive-ness/  recovery orientation/observation | Professional identifies resources of the patient and coping strategies that can be strengthened in the treatment plan. | Professional integrates internal and external resources into the treatment plan, including interpersonal relationships. | Professional identifies resources of the patient and coping strategies that can be strengthened in the treatment plan. | Professional integrates internal and external resources into the treatment plan, and involves support persons in the treatment. |
| 35./  38./  36./  37. | Treatment/  Responsive-ness/  sensitivity | Professional adjusts the intervention when a patient is not improving or deteriorating, rather than blaming the patient. | Professional adjusts the psychological intervention when a patient is not improving or deteriorating, rather than blaming the patient. | Professional adjusts the pharmacological intervention when a patient is not improving or deteriorating, rather than blaming the patient. | Professional adjusts the pharmacological intervention when a patient is not improving or deteriorating, rather than blaming the patient. |
| 36./  39./  37./  38. | Treatment/  assessment/observation  ethics/  reflection | Professional brings up ethical issues in case discussion, such as risk concerns, concerns about the fit of intervention or limits of competency, or conflicts of interest. | Professional brings up ethical issues in case discussion, such as risk concerns, concerns about the fit of intervention or limits of competency, or conflicts of interest. | Professional brings up ethical issues in case discussion, such as risk concerns, concerns about the fit of intervention or limits of competency, or conflicts of interest. | Professional brings up ethical issues in case discussion, such as risk concerns, concerns about the fit of intervention or limits of competency, or conflicts of interest. |
| 37./  40./  39. | Treatment/  hypotheses/  responsive-ness | Professional suggests explanations for the patient’s suffering and adapts them based on what they observe in session. | Professional suggests hypotheses about the patient’s suffering that can be adapted based on the elaboration with the patient. |  | Professional suggests hypotheses about the patient’s suffering that are adapted based on continued assessment. |
| 38./  41./  40. | Treatment/  Theoretical knowledge/  responsiveness | Professional can define the next steps of an intervention based on the patient’s priorities (current concerns of the patient, basic needs, …) and suffering. | Professional can prioritise appropriate intervention techniques based on functional impairment and evidence about “what works” for mental health conditions, and the needs of the patient. |  | Professional can define a treatment plan based on evidence, carefully weighing the costs and benefits of a pharmacological intervention. |
| 39./  42./  41. | Treatment/  theoretical knowledge/  responsive-ness | Professional can discuss reasons for choosing these steps, shows theoretical grasp of common counselling concepts (e.g. active listening, psychoeducation). | Professional can discuss intervention based on psychological theory (about human development, about psychopathology, …), has a clear rationale for it and adjusts it. |  | Professional has a clear rationale for intervention based on management plans for mental disorders and can adjust it. |
| 40./  43./  42. | Assessment/  Responsive-ness/obser-vation/direc-tiveness | Professional can discuss readiness of the patient for change, and things that may facilitate change or hold the patient back. | Professional can discuss readiness of the patient for change, and adapt the intervention appropriately. |  | Professional can discuss readiness of the patient for change, and adapt the intervention appropriately. |
| 41./  44. | Treatment/  reflection | Professional can discuss the relationship with the patient, what is easy/difficult about it and distinguish between their own and the patient’s feelings. | Professional can discuss the relationship with the patient, and reflect on how their own reaction to the patient affects the therapeutic interaction. |  |  |
| **C. Clinical Files (file review)** | | | | | |
| 42./  45./  38./  43. | General/  documenta-tion | Professional uses the appropriate clinical forms after each consultation. | Professional fills the appropriate clinical forms completely after each consultation. | Professional uses the appropriate clinical forms after each consultation. | Professional uses the appropriate clinical forms completely after each consultation. |
| 43./  46./  39./  44. | Assessment/  structure/  theoretical knowledge | Professional summarises the patient’s story in terms of presenting complaint and symptoms reported. | Professional is specific in descriptions of intensity, frequency, evolution of symptoms. | Professional summarises the patient’s story in terms of presenting complaint and symptoms reported. | Professional is specific in descriptions of intensity, frequency, evolution of symptoms. |
| 44./  47./  40./  45. | Assessment/  observation/  reflection | Professional documents own observations of the patient and makes a note when worried about a risk issue. | Professional documents own observations of the patient (affect, appearance, language, interaction). | Professional documents own observations of the patient and makes a note when worried about a risk issue. | Professional documents own observations of the mental status of the patient. |
| 45./  48./  41./  46. | Treatment/  structure/  theoretical knowledge | Professional writes a treatment plan for follow-up, identifying main goals of the counselling. | Professional writes a treatment plan for follow-up appropriate to the patient needs and clinical presentation. | Professional writes a treatment plan for follow-up, identifying the main medical needs. | Professional writes a treatment plan for follow-up appropriate to the patient needs and clinical presentation. |
| 46./  49./  42./  47. | Treatment/  responsibili-ty/rapport | Professional provides regular follow-up, making documented efforts to contact patients not coming. | Professional provides regular follow-up, making documented efforts to contact patients not coming. | Professional provides regular follow-up, making documented efforts to contact patients not coming. | Professional provides regular follow-up, making documented efforts to contact patients not coming. |
| 43./  48. | Treatment/  medical/  continuity |  |  | Professional documents all prescriptions clearly (including amount given to patient). | Professional documents all prescriptions clearly (including amount given to patient). |
| 44./  49. | Treatment/  medical/  risk |  |  | Professional gives the appropriate amount of a drug in relation to frequency of appointments, avoiding gaps and/or supplying large quantities with insufficient monitoring. | Professional gives the appropriate amount of a drug in relation to frequency of appointments, avoiding gaps and/or supplying large quantities with insufficient monitoring. |
| **D. Outside sessions (everyday observations, MDT work)** | | | | | |
| 47./  50./  45./  50. | Multidisci-plinary work | Professional communicates about the treatment plan with other members of the team. | Professional communicates about the treatment plan with other members of the team. | Professional communicates about the treatment plan with other members of the team. | Professional communicates about the treatment plan with other members of the team. |
| 48./  51./  46./  51. | Multidiscipli-nary work/  responsibi-lity | Professional makes appropriate referrals, makes active efforts to understand the network. | Professional makes appropriate referrals, makes active efforts to understand the network. | Professional makes appropriate referrals, makes active efforts to understand the network. | Professional makes appropriate referrals, makes active efforts to understand the network. |
| 49./  52./  47./  52. | Multidiscipli-nary work/  reflection | Professional gives feedback to others in a constructive, respectful way. | Professional gives feedback to others in a constructive, respectful way. | Professional gives feedback to others in a constructive, respectful way. | Professional gives feedback to others in a constructive, respectful way. |
| 50./  53./  48./  53. | Multidiscipli-  nary work/  reflection/  openness/  learning | Professional is able to integrate feedback on the patient care. | Professional is able to integrate feedback on the patient care and adjust own practice. | Professional is able to integrate feedback on the patient care. | Professional is able to integrate feedback on the patient care and adjust own practice. |
| 51./  54./  49./  54. | Responsibili-ty | Professional accepts responsibility for own actions. | Professional accepts responsibility for own actions. | Professional accepts responsibility for own actions. | Professional accepts responsibility for own actions. |
| 52./  55./  50./  55. | Reflection/  openness/  learning | Professional seeks supervision when he/she needs support. | Professional seeks supervision when he/she needs support. | Professional seeks supervision when he/she needs support. | Professional seeks supervision when he/she needs support. |
| 53./  56./  51./  56. | Responsibi-lity/  Professio-nalism | Professional collects and submits all required data in a timely and complete manner. | Professional collects and submits all required data in a timely and complete manner. | Professional collects and submits all required data in a timely and complete manner. | Professional collects and submits all required data in a timely and complete manner. |
| 54./  57./  52./  57. | Ethics | Professional respects confidentiality of the patient and family, and treats their information sensitively. | Professional respects confidentiality of the patient and family, and treats their information sensitively. | Professional respects confidentiality of the patient and family, and treats their information sensitively. | Professional respects confidentiality of the patient and family, and treats their information sensitively. |

# Structure and Weighting of the Item Pool

**Overview**: Number of Items per level and category (as an estimate of weighting of items).

| **Category:** | **Counselling - Level 1:** | **Counselling - Level 2:** | **mhGAP/psychiatric-**  **Level 1:** | **mhGAP/psychiatric – Level 2:** |
| --- | --- | --- | --- | --- |
| *A. Observed Transversal Skills* | *13* | *13* | *13* | *13* |
| 1. Communication with the patient (live) | 9 | 9 | 9 | 9 |
| 2. Organisation of session (live) | 4 | 4 | 4 | 4 |
| *B. Observed Technical Skills* | *28* | *32* | *24* | *29* |
| 1. In session (live supervision) | 16 | 20 | 17 | 18 |
| 2. In case discussion (non-live) | 12 | 12 | 7 | 11 |
| *C. Clinical Files (file review)* | *5* | *5* | *7* | *7* |
| *D. Outside sessions (everyday observations, MDT work)* | *8* | *8* | *8* | *8* |
| **TOTAL** | **54** | **58** | **52** | **57** |

**Overview**: Frequency of codes/assumed latent constructs.

| **Assumed construct:** | **Overall Frequency:** |
| --- | --- |
| **Treatment (superordinate)** | **32** |
| **Assessment (superordinate)** | **10** |
| Therapeutic techniques | 12 |
| Responsiveness | 9 |
| Theoretical knowledge (MH) | 8 |
| Medical | 7 |
| Reflection | 6 |
| Risk | 6 |
| Structure | 6 |
| Observation | 5 |
| Rapport | 5 |
| Empathy | 4 |
| Directiveness | 5 |
| Responsibility | 4 |
| Multidisciplinary work | 4 |
| Recovery orientation | 4 |
| Organisation | 3 |
| Communication | 4 |
| Differentiation | 3 |
| Sensitivity | 4 |
| Ethics | 3 |
| **Diagnosis (superordinate)** | **2** |
| Boundaries | 2 |
| Hypotheses | 2 |
| Trauma | 2 |
| Openness | 2 |
| Learning | 2 |
| Continuity | 2 |
| Crisis | 1 |
| Groups | 1 |
